# Non-pharmacological interventions and quality of life in female survivors of hormone receptor-positive breast cancer receiving adjuvant endocrine therapy: A systematic review

**DOI:** 10.23938/ASSN.1176

**Published:** 2026-08-27

**Authors:** Ariana Arias-Martínez, Paula Escalada-Hernández, Gustavo Adolfo Pimentel-Parra, Cristina García-Vivar, Nelia Soto-Ruiz

**Affiliations:** 1 Public University of Navarre (UPNA). Department of Health Sciences. Pamplona, Navarra. Spain. Public University of Navarre (UPNA) Department of Health Sciences Pamplona Navarra Spain; 2 Navarre Institute of Health Research (IdiSNA). Pamplona, Navarra. Spain. Navarre Institute of Health Research (IdiSNA) Pamplona Navarra Spain

**Keywords:** Cancer Survivors, Breast Neoplasms, Antineoplastic Agents, Hormonal, Quality of Life, Systematic Review, Supervivientes de Cáncer, Cáncer de mama, Antineoplásicos Hormonales, Calidad de Vida, Revisión Sistemática

## Abstract

**Background:**

Hormone receptor-positive breast cancer is treated with endocrine therapies to reduce recurrence and mortality. These therapies have side effects that negatively affect quality of life (QoL). We aim to synthesize the available evidence on the impact of non-pharmacological interventions on QoL and its specific domains (physical, psychological, social, and spiritual) in female breast cancer survivors undergoing endocrine therapies.

**Methods:**

A systematic review following the Cochrane and PRISMA guidelines was registered in PROSPERO (CRD42024524238). Studies published from January 2013 to October 2025 were retrieved from PubMed, the Cochrane Library, Scopus, and Web of Science. Randomized controlled trials evaluating QoL in female with BC aged over 18 years, and undergoing endocrine therapies were included. Due to methodological and clinical heterogeneity, a narrative synthesis was performed instead of a meta-analysis. The RoB2 tool was used to assess the risk of bias.

**Results:**

Seven studies (with 713 participants) were included. Non-pharmacological interventions such as physical exercise, hydrotherapy, physiotherapy, and health promotion programs were analysed. Physical exercise interventions showed significant improvement in overall QoL and specific domains.

**Conclusions:**

Non-pharmacological interventions show variable effects on QoL in female breast cancer survivors undergoing endocrine therapies. While physical exercise appears promising, the high methodological heterogeneity and limited evidence preclude definitive conclusions. Future research should focus on standardized approaches.

## RESUMEN

**Fundamento:**

La terapia endocrina (TE) es el tratamiento del cáncer de mama con receptores hormonales; reduce el riesgo de recidiva y la mortalidad, pero presenta efectos secundarios que disminuyen la calidad de vida (CdV). El objetivo es sintetizar la evidencia sobre el impacto de las intervenciones no farmacológicas sobre la CdV y sus dimensiones específicas (física, psicológica, social y espiritual) en mujeres supervivientes de cáncer de mama (SCM).

**Métodos:**

Revisión sistemática siguiendo las directrices Cochrane y PRISMA, registrada en PROSPERO (CRD42024524238). Se recuperaron estudios publicados entre enero de 2013 y octubre de 2025 en PubMed, Cochrane Library, Scopus y Web of Science. Se incluyeron ensayos clínicos aleatorizados que evaluaban la CdV de SCM mayores de 18 años y sometidas a TE. Debido a la heterogeneidad metodológica y clínica, se realizó una síntesis narrativa y no un meta análisis. El riesgo de sesgo se evaluó con la herramienta RoB 2.

**Resultados:**

Se incluyeron siete estudios, con 713 participantes. Se analizaron intervenciones no farmacológicas como ejercicio físico, hidroterapia, fisioterapia y programas de promoción de la salud. Las intervenciones basadas en ejercicio físico mejoraron significativamente la CdV, tanto global como algunas dimensiones.

**Conclusiones:**

Los resultados de las intervenciones no farmacológicas en SCM sometidas a TE sobre la CdV son variables. Aunque el ejercicio físico parece una intervención prometedora, la elevada heterogeneidad metodológica y la limitada evidencia disponible impiden establecer conclusiones definitivas. Las investigaciones futuras deberían centrarse en enfoques estandarizados.

## INTRODUCTION

Breast cancer (BC) is one of the most diagnosed cancers worldwide and ranks first among cancers in women^1^^-^^3^. Although the global incidence of BC continues to increase, improvements in screening programs and advances in treatment have contributed to a decrease in mortality^4^^,^^5^ and an increase in survival^3^.

BC has one of the highest five-year survival rates, exceeding 90% in the early stages^6^. However, the risks of recurrence and adverse effects continue after the end of the primary treatment -including chemotherapy, radiotherapy, immunotherapy and/or surgery- and a second episode of BC can occur at any time^7^^,^^8^. Endocrine therapy (ET) has revolutionized the treatment of hormone receptor-positive (HR+) BC, resulting in significant reductions in recurrence and mortality^9^^-^^11^.

ET is indicated for women with hormone HR+ tumours, namely, oestrogen receptor positive tumours (ER+), progesterone receptor-positive tumours (PR+), or both receptor-positive tumours^12^^-^^14^. These types of cancer constitute approximately 85% of diagnosed BC cases^12^^,^^13^. ET works by interfering with signalling through oestrogen receptors, as is the case with tamoxifen, or by reducing oestrogen production, as is the case with aromatase inhibitors (e.g., anastrozole, letrozole, and exemestane)^12^^-^^14^. This way, tumour growth is inhibited through the deprivation of these hormones induced by these therapies^15^.

ET is usually indicated for at least 5 years^16^ and may be extended to 10 years in women at higher risk of recurrence who can tolerate the treatment^17^. However, several side effects are associated with the use of ET. Tamoxifen is often associated with vaginal bleeding, endometrial cancer, and venous thromboembolic events, whereas aromatase inhibitors can cause arthralgia or, more rarely, bone fractures^18^. Both types of drugs can cause vasomotor and/or vulvovaginal symptoms, such as hot flushes, night sweats, vaginal dryness, and sexual dysfunction, as well as physical deterioration, joint pain and dysfunction, cardiovascular disease, or psychological disorders^19^^,^^20^. These side effects can significantly affect quality of life (QoL) and its various dimensions, including physical, emotional, cognitive, and social functioning.

As a result, some women have difficulty adhering to treatment^19^^,^^21^, with approximately half of women taking less than 80% of the prescribed dose^10^^,^^22^ and non-adherence rates of 16% have even been reported^23^.

Owing to the complexity of the long-term side effects of ET, it is essential to acquire greater knowledge to provide better care and monitoring of QoL in female breast cancer survivors (BCS)^13^^,^^24^^-^^26^.

Consequently, several pharmacological and non-pharmacological interventions have been implemented to mitigate the side effects associated with ET. However, it is necessary to determine whether these interventions contribute to improve QoL in BCS undergoing ET^27^. A systematic review performed in 2020 on pharmacological and non-pharmacological interventions for symptoms and QoL in BCS undergoing ET concluded that, owing to the heterogeneity and inconsistency of results across studies, firm conclusions could not be drawn regarding which type of intervention was most optimal^28^. In 2021, Franzoi *et al*. synthesized pharmacological, non-pharmacological, and complementary medicine strategies for the management of isolated symptoms associated with endocrine therapy in a narrative review^27^. They highlighted that physical exercise and cognitive behavioural therapy had the most robust and consistent evidence for fatigue and multiple symptoms^27^. Considering these findings, a gap in knowledge persists regarding the specific effect of non-pharmacological interventions on QoL, based on an updated systematic methodology.

Therefore, the objective of this study is to identify, evaluate, and synthesize the available scientific evidence on the impact of non-pharmacological interventions on QoL and its specific domains (physical, psychological, social, and spiritual) in female BCS undergoing ET.

## MATERIAL AND METHODS

### Design

A systematic review was carried out. The review followed the Cochrane *Handbook for Systematic Reviews of Interventions*^29^, and the data were reported according to the Preferred Reporting Items for Systematic Review and Meta-Analysis (PRISMA)^30^. The protocol of the systematic review was registered in the PROSPERO International Prospective Register of Systematic Reviews with the identifier CRD42024524238. Although the original protocol encompassed both pharmacological and non-pharmacological interventions, the scope was subsequently restricted to non-pharmacological interventions alone based on the results of the preliminary search. This modification constitutes a deviation from the originally registered protocol. Thus, this systematic review aimed to answer the following research question: Which non-pharmacological interventions are effective in improving quality of life in female BCS undergoing ET compared with standard care or no intervention?

### Eligibility criteria

The PICO model was used to define the inclusion and exclusion criteria. Randomised controlled trials (RCTs) were included according to the following criteria:

- Population (P): women who were survivors of BC, currently receiving ET, and aged over 18 years. To ensure a homogeneous sample, eligible participants must have completed their primary cancer treatments (surgery, chemotherapy, and/or radiotherapy) and be receiving ongoing ET. Both premenopausal and postmenopausal women were eligible for inclusion. Although 0.5% to 1% of all BC cases diagnosed annually occur in men, and ET is an important treatment modality, men were not included in this study because their needs may differ from those of women, especially in relation to the psychosocial dimension.
- Intervention (I): any type of non-pharmacological intervention aimed at improving QoL in women receiving ET. Non-pharmacological interventions are science-based, non-invasive interventions for human health. They aim to prevent, treat, or cure health problems. Examples might include psychological, physical health, nutritional, digital health interventions, among other.
- Comparison (C): not limited to any type of comparator. Studies were included if the intervention was compared with placebo interventions, usual care/standard care, active comparators, or no intervention.
- Outcomes (O): The primary outcome was QoL (global or overall score) assessed using validated instruments. Secondary outcomes were QoL domains: physical, psychological, social, and spiritual. Furthermore, other potential areas of QoL, such as sexual functioning, were included. Studies were eligible regardless of whether QoL was designated as a primary or secondary endpoint in the original trial design.

Therefore, the inclusion criteria established for the selection of the studies were: 1) randomized controlled trials; 2) including women who were survivors of BC; 3) previous primary cancer treatment with surgery and/or chemotherapy, radiotherapy, or immunotherapy; 4) aged over 18 years; 5) currently receiving ET; and 6) non-pharmacological interventions for which the effects on QoL and its specific domains were evaluated. Studies were excluded if they described patients receiving palliative treatment.

### Search strategy

Bibliographic searches of the literature were performed in the PubMed, Cochrane Library, Scopus, and Web of Science databases because of their recognized quality, international coverage and relevance in identifying high-impact scientific literature in biomedical and health disciplines.

The search strategy was adapted specifically for each database. Boolean operators (AND, OR) were used together with the descriptors and related keywords: (“Cancer survivor” OR “cancer survivorship” OR “breast cancer survivor” OR “breast neoplasm survivor”) AND (“breast neoplasm” OR “breast tumour” OR “breast cancer” OR “breast carcinoma”) AND “hormone replacement therapy” OR “hormone therapy” OR “endocrine therapy” OR tamoxifen OR “aromatase inhibitor therapy” OR letrozole OR anastrozole OR exemestane). Appendix I presents in detail the search strategies used for the different databases. The initial literature search was conducted in February 2024, limiting the publication period to the preceding 10 years (January 2013 to December 2023). Subsequently, to include the most recent evidence, a search update was performed in October 2025. Therefore, the total period evaluated in this review spanned from January 2013 to October 2025. The literature search was restricted exclusively to randomized controlled trials. Trial records, grey literature databases, and backward/forward citation screening were not formally searched, nor were authors contacted for additional data; all search limitations are explicitly declared. Language restrictions were applied to articles published in English and Spanish. The search results were exported to the Rayyan^31^ platform to manage the results of the systematic review.

### Selection of studies

Two independent investigators (AAM, NSR) performed the selection process. A third reviewer (CGV) resolved discrepancies in study selection. Initial filtering was conducted to remove duplicate records. The next step in screening involved reading the title and abstract of the studies. Finally, the full texts were assessed according to the eligibility criteria.

### Quality of the studies

The risk-of-bias of the included studies was evaluated by two independent reviewers using the Cochrane Risk of Bias 2.0 v7^32^ (RoB 2.0) assessment tool. The risk of bias was categorized as *low*, *some concerns*, or *high* and was assessed across five domains: D1, bias arising from the randomization process; D2, bias due to deviations from intended interventions; D3, bias to missing outcome data; D4, bias in measurement of the outcome; and D5, bias in selection of the reported result. The certainty of the evidence was assessed according to the authors´ judgement using the GRADE methodology. As the included studies were randomized controlled trials, the evidence initially started at *high* certainty. Five domains were assessed for potential downgrading: risk of bias, inconsistency, indirectness, imprecision, and publication bias. The certainty of the evidence was downgraded by one level for serious concerns or by two levels for very serious concerns. The three upgrading domains -large magnitude of effect, dose-response gradient, and plausible residual confounding that would reduce the observed effect- were also considered. Accordingly, the overall certainty of evidence for each outcome was classified into four levels: high (4), moderate (3), low (2), or very low (1).

### Data extraction

Two independent investigators (AAM, NSR) carried out data extraction from the selected studies. In cases of disagreement or discrepancies, a third reviewer (CGV) was consulted to reach consensus. One table containing the main characteristics of the included studies was prepared, including; 1) author, year, and country; 2) design, sample size, and age; 3) intervention and comparator (IG/CG); and 4) measurement instruments, main results, and effect size.

### Data analysis

Initially, a quantitative synthesis involving a meta-analysis was planned. However, during data extraction, significant clinical and methodological heterogeneity was identified among the included studies. The interventions exhibited substantial heterogeneity in terms of the types of non-pharmacological protocols, duration, and intensity, and the variety of QoL measurements instruments. This substantial variability precluded conducting a meta-analysis of QoL and its domains. Consequently, the results were presented using a narrative approach.

The narrative synthesis was structured according to four categories: type of intervention (physical exercise, hydrotherapy, physiotherapy, and health promotion), QoL measurement instruments, intervention effects on QoL, and QoL domains assessed.

To evaluate the magnitude and clinical relevance of the interventions beyond statistical significance (*p*-values), effect sizes were systematically analysed. For trials that did not explicitly report effect sizes in their original text, these were calculated manually from the published descriptive data (means, SDs, or 95% CIs)^33^. Specifically, Cohen’s *d* or Hedges’ *g* were used depending on sample sizes, and their magnitudes were interpreted according to standard criteria: small (≥ 0.20), medium (≥ 0.50), or large (≥ 0.80).

## RESULTS

### Study selection and risk of bias assessment

**Figure 1:**
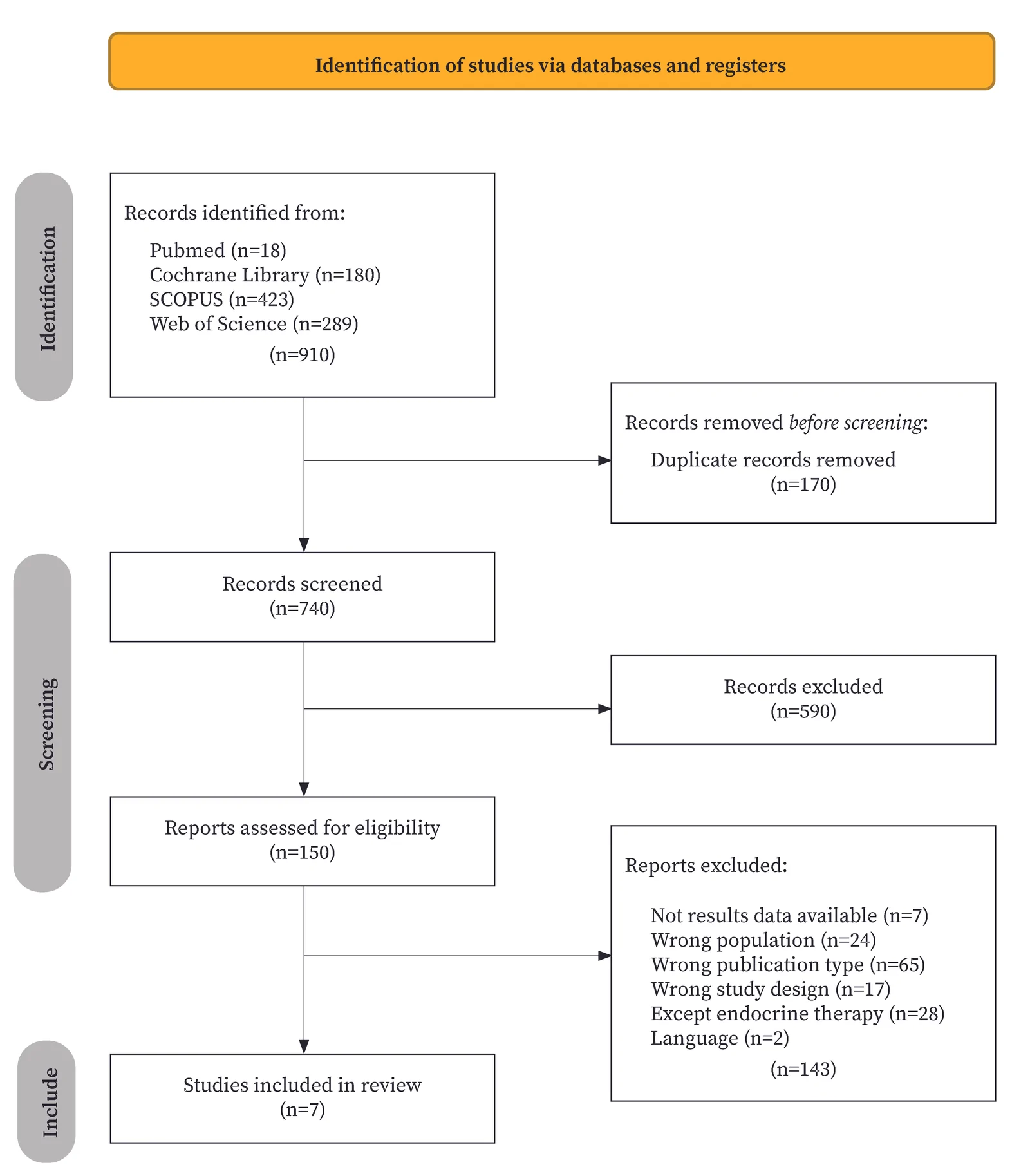
PRISMA flow diagram.

A total of 910 studies were identified, and 170 duplicates were removed. After removal of duplicates, 740 studies were screened based on title and abstract, leading to the exclusion of 590 studies. The full texts of the remaining 150 articles was assessed according to the inclusion and exclusion criteria. Ultimately, seven articles met the selection criteria. The selection process was exhaustively recorded, allowing the elaboration of a PRISMA flow diagram (Fig. 1).

**Figure 2:**
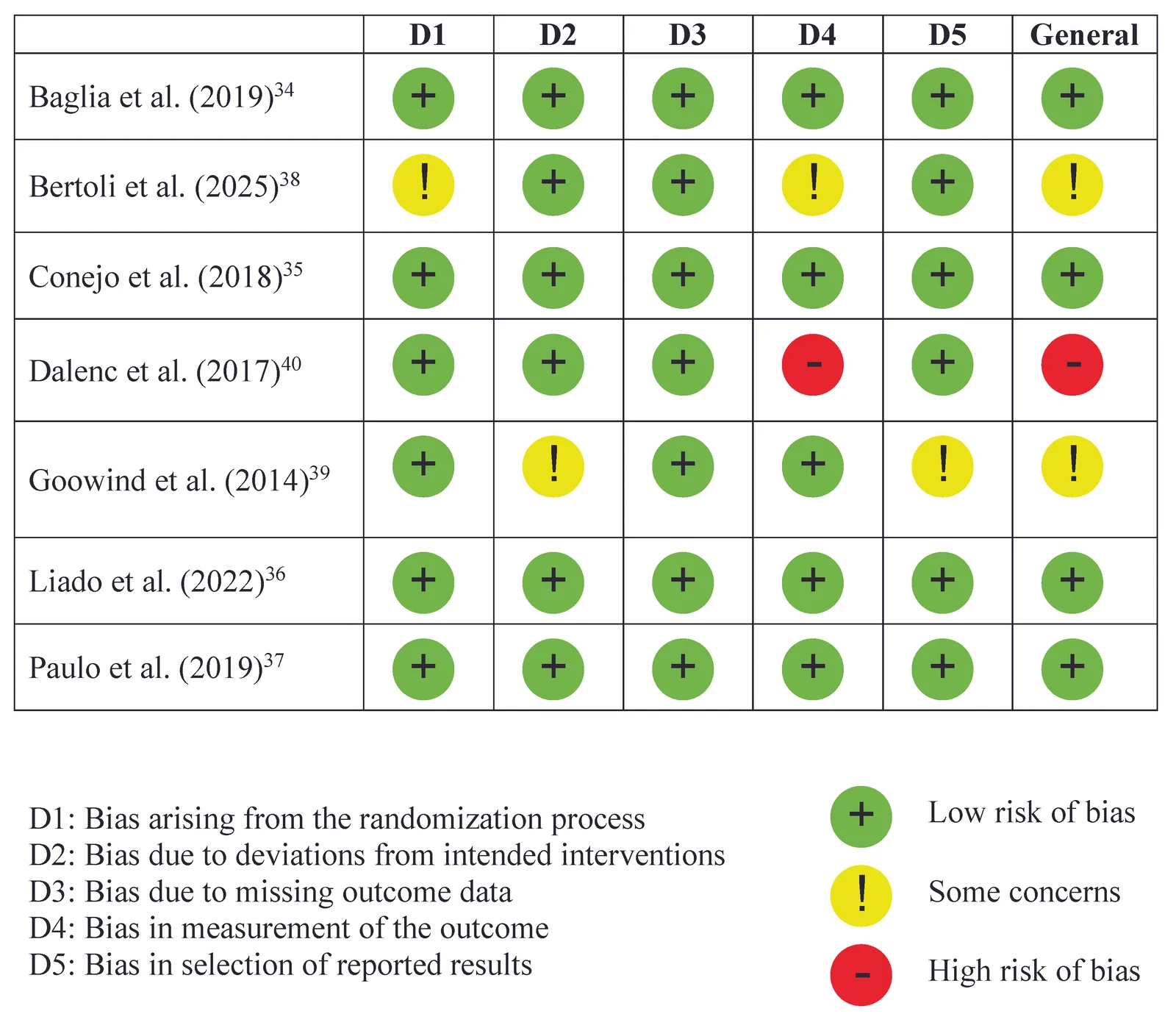
Risk of bias using the RoB 2 tool.

Four trials^34^^-^^37^ (57.14%) had a *low* risk of bias across all dimensions, reflecting high methodological quality. However, specific domain vulnerabilities were identified in the remaining studies. Two of them^38^^,^^39^ (28.57%) raised *some concerns* overall, owing to potential uncertainties in D1 and D2. The remaining study (14.28%) presented an overall *high* risk of bias, driven by critical concerns in D4 (measurement of the outcome), as the unblinded, patient-reported nature of QoL assessment in this trial introduced a potential risk of detection bias^40^ (Fig. 2).

### Study and intervention characteristics

The included studies were conducted across six countries: Brazil (n=2)^37^^,^^38^, Canada^39^, China^36^, Spain^35^, the United States^34^, and France^40^. Sample sizes ranged from 36^37^ to 338^39^ female BCS, yielding a total sample of 713 participants with a mean age of 59.67 years (± 5.24) (Table 1).

Most of the trials, 57.1% (n = 4), were based on structured physical exercise protocols^34^^,^^36^^-^^38^. These included aerobic and strength training programs^34^, combined aerobic-resistance exercise^37^, Baduanjin mind-body practice^36^, and a structured mat Pilates program^38^. These interventions differed primarily in duration and frequency, but were all conducted under professional supervision.

The remaining three studies implemented diverse non-pharmacological interventions. These included hydrotherapy under specialist supervision^40^, a physiotherapy approach using neuromuscular taping for pain relief^35^, and a 24-month health promotion program delivered via email and mobile devices, providing information and guidance on diet and physical activity^39^.

Regarding the control groups (CG), although no initial limitations were applied to the comparators during the screening phase, the synthesis of the seven trials revealed two distinct methodological approaches: passive usual care^34^^,^^36^^,^^40^ and active controls or sham placebos. including mindfulness/meditation sessions^38^, simulated neuromuscular taping^35^, healthy lifestyle e-mail guidelines^39^, and bi-weekly stretching programs^37^.

**Table 1:** Main characteristics of the selected randomized controlled trials included in the study

Author / Year / Country	Sample size	Intervention and comparator	Measurement instruments (Main results)
	Age	Setting and supervision	Effect size (Cohen’s d / Hedges’ g)
Baglia et al.^34^	n=121	IG: 12-month gym-based supervised physical activity program (150 minutes of aerobic exercise + two strength training sessions weekly)	IG had significantly greater improvements compared to CG:
(2019)	IG=61; CG=60	CG: Usual care (monthly telephone contact discussing health education topics)	- FACT-G: general score (+8.0 vs +1.2); physical well-being (2.4 vs -1.2), functional well-being (2.6 vs 0.1)
EEUU	Postmenopausal BCS receiving AIs	Community: Gymnasium	- FACT-B: total score (10.2 vs 2.0).
	IG: 62.0±7.0	Individual	- SF-36: physical functioning (6.3 vs -1.1), social role functioning (6.2 vs 1.4) and mental functioning: (4.0 vs 0.7)
	CG: 60.5±7.0		Standardized ES: 0.46 (FACT-B and FACT-G; metric not specified
Bertoli et al.^38^	n=40	IG: 24-week structured mat Pilates program (three 60-minute sessions weekly)	Significant improvements observed in the IG compared to the CG in:
(2025)	IG=21; CG=19	CG: bi-weekly intervention comprising self- awareness exercises, relaxation techniques, and mindfulness meditation	- SF-36: global health status (11.5 vs 5.9 at 24 weeks), social aspects (+15.1 vs -11.6 group x time), mental health (7.0 vs -2.2 at 12 weeks).
Brasil	BCS receiving HT	Community: Exercise-room	- EORTC QLQ-C30 and EORTC QLQ-BR23: ns
	IG: 55.2±7.6	Group	Large ES favouring the IG: EORTC QLQ-C30 (emotional functioning, d=1.71), SF-36: global health status (d=3.76), social aspects (d=2.49), mental health (d=1.56)
	CG: 55.4±6.6		
Conejo et al.^35^	n=40	IG: neuromuscular taping (TNM) for 5 weeks in painful areas	IG achieved significantly greater improvements compared to the CG in EORTC QLQ-C30: global health status (22.92 vs 5.83)
(2018)	IG=20; CG=20	CG: simulated neuromuscular taping	Remaining functional scales: ns
Spain	BCS receiving ET	Hospital: Outpatient-clinic	ES*: global health status (g=1.73), role functioning (g=1.22), cognitive functioning (g=0.81)
	Total: 66.30 (50-82)	Individual	
	IG: 67.80 (50-82)		
	CG: 64.80 (54-81)		
Dalenc et al. ^40^	n=70	IG: 3-weeks hydrotherapy at a thermal center (daily regimen alternating baths and thermal water showers, oral intake of thermal water, wrapping with emollient balm, specific massages performed and aesthetic care in the form of make-up)	EORTC QLQ-BR23 scores significantly improved in the IG.
(2018)	IG=35; CG=33	CG: Usual care	Cancer-related QoL and health status
France	BCS receiving AHT, with persistent dermatologic adverse events	Thermal-facility: Spa-center	assessed (EORTC QLQ-C30): ns
	IG: 53.0±7.2	Group	Not calculable ES (descriptive data not shown)
	CG: 51.5±10.9		
Goodwin et al. ^39^	n=338	IG: Telephone-based individualized intervention for 24 months; 19 standardized calls by coaches to achieve weight loss goals, diet modifications, a gradual increase in moderate-intensity physical activity, along with motivation and behavioral strategies)	Significantly greater improvement favouring the IG in:
(2014)	IG=171; CG: 167	CG: Mail-based intervention that included information on healthy lifestyles (nutritious diets, physical exercise, recommendations for adherence to treatment and general medical issues)	- SF-36: Physical component (4.4 vs 4.1).
Canada	Postmenopausal BCS receiving AHT	Community: Home	- EORTC QLQ-C30: Physical functioning (1.01 vs 0.64).
	IG: 61.6±6.7	Individual by phone	ES*: SF-36: physical component (d=0.221), EORTC QLQ-C30: physical functioning (d=0.228).
	CG: 60.4±7.8		
Liao et al.^36^	n=72	IG: Baduanjin exercise program for 12 weeks. Weekly two supervised sessions:10-minute warm-up, 70 minutes of Baduanjin practice and 10 minutes cool-down	IG showed significantly greater improvements compared to the CG in:
(2022)	IG=36; CG=36	CG: Usual care and evading Baduanjin exercises	- EORTC QLQ-C30: global QoL (12.39 vs -2.31), physical functioning (8.48 vs -3.66), role functioning (6.88 vs -1.29), emotional functioning (9.42 vs -0.23), cognitive functioning (9.21 vs -0.83)
China	BCS receiving AIs ≥6 months	Hospital: Exercise-room	Large ES*on global QoL (g=0.97) and physical functioning (g=1.03)
	IG: 53.12±7.02	Group	
	CG:54.63±8.44		
Paulo et al. ^(^^37^	n=36	IG: Combined exercise training program for 9 months. Weekly 3 sessions: 40 minutes of machine-based resistance training, and 30 minutes of aerobic training	IG showed significantly greater improvements compared to the CG (significant time x group interactions) in:
(2019)	IG=18; CG=18	CG: Invitation to participate in stretching and relaxation activities, scheduled twice a week, with sessions of 45 minutes	- SF-36: General health perception (11.5 vs 3.5), physical functioning (8.1 vs 4.1), social functioning (17.5 vs 1.8)
Brazil	Postmenopausal BCS undergoing AIs	Community: Fitness-laboratory	- EORTC QLQ-C30: Role functioning (28.3 vs 5.61).
	IG: 63.2±7.1	Group	- EORTC QLQ-BR23: ns in sexual functioning
	CG: 66.6±9.6		Large ES favouring the exercise group in several variables: SF-36: physical functioning (d=1.32), general health (d=1.14), and social functioning (d=1.10); EORTC QLQ-C30: role functioning (d=1.05)

BCS: breast cancer survivors; AHT: adjuvant hormonal therapy; AIs: aromatase inhibitors therapy; CG: control group; ES: effect size (large if d≥0.8); ET: endocrine therapy HT: hormonal therapy; IG: intervention group; ns: not statistically significant differences; *: this effect size parameter was not explicitly reported in the original study and was calculated manually by this review authors using the published descriptive statistics. EORTC-QLQ-C30: European Organization for the Research and Treatment of Cancer Quality of Life Questionnaire - Core 30; EORTC-QLQ-BR23: European Organization for the Research and Treatment of Cancer Quality of Life Questionnaire - Breast Cancer; FACT-G: Functional Assessment of Cancer Therapy - General; FACT-B: Functional Assessment of Cancer Therapy - Breast; SF-36: 36-Item Short Form Survey.

### QoL measurement instruments

To evaluate QoL, six studies^35^^-^^40^ (85.71%) used the European Organization for the Research and Treatment of Cancer Quality of Life Questionnaire Core 30 (EORTC-QLQ-C30), a cancer-specific questionnaire structured into several functional areas and symptom scales. In three studies^37^^,^^38^^,^^40^, QoL was additionally assessed using a BC-specific instrument, the EORTC-QLQ-BR23, and/or a generic instrument, the 36-Item Short Form Survey (SF-36). The remaining study^34^ employed, in addition to the SF-36 and Functional Assessment of Cancer Therapy - General (FACT-G), a cancer-specific instrument (FACT-B: Functional Assessment of Cancer Therapy - Breast). Not all studies reported global QoL scores. While one study focused exclusively on specific QoL domains^40^, the other assessed both specific domains and the global score^34^^-^^39^.

### Effects of interventions on overall QoL and general health perception

The four studies^34^^,^^36^^-^^38^ evaluating physical exercise interventions were heterogeneous regarding measurement instruments, intervention type (aerobic exercise combined with strength training^34^ or resistance exercise^37^, a mat Pilates program^38^, or Baduanjin^36^, methodology, and findings.

Two studies reported statistically significant improvements in overall/global QoL in the intervention group (IG). Baglia *et al*. (2019)^34^ evaluated a 12-month supervised aerobic and resistance exercise program, observing better results in the IG on both FACT-G (8.0 vs 1.2) and FACT-B, with standardized effect sizes of 0.46; the authors did not report the specific effect size metric (Cohen’s *d* or Hedges’ *g*)^34^ (Table 1). Liao *et al*. (2022)^36^ demonstrated positive effects of Baduanjin exercises in the IG, with a mean increase of 12.39 points in global QoL measured by EORTC QLQ-C30, whereas the CG declined. This improvement reached or exceeded the minimal important difference established in previous clinical studies^36^.

The remaining two studies provided data regarding global health status perception. Paulo *et al*. (2019)^37^ reported significant time x group interactions favouring the IG on the SF-36, with a large effect size, after a 9-month combined aerobic and resistance training programme; however, no significant differences were observed in the global health status domain of the EORTC QLQ-C30. Similar instrument-dependent patterns were found in the mat Pilates intervention conducted by Bertoli *et al*. (2025)^38^. When global health status was assessed using the generic SF-36 questionnaire, the IG achieved statistically significant improvements compared with the CG at 24 weeks (11.5 vs 5.9), with a large effect size, whereas the EORTC QLQ-C30 did not reveal statistically significant inter-group differences.

The three remaining non-pharmacological interventions^35^^,^^39^^,^^40^ exhibited highly divergent results. Conejo *et al*. (2018)^35^ evaluated a physiotherapy intervention using neuromuscular taping; the results showed significant differences favouring the IG in EORTC QLQ-C30 global health status, with a large effect size. Regarding the impact of the hydrotherapy intervention on QoL, Dalenc *et al*. (2018) found differentiated patterns of improvement depending on the questionnaire used: while QoL scores on the EORTC QLQ-BR23 improved significantly more in the IG compared to the CG, no statistically significant differences were observed when health status was assessed using the core EORTC QLQ-C30 questionnaire^40^. Finally, Goodwin *et al*. (2014)^39^ evaluated a health-promotion intervention (baseline QoL values were not reported); at 24 months, EORTC QLQ-C30 overall QoL showed no statistically significant inter-group differences.

### Effects of interventions on the physical domain

Across the four studies analysing physical exercise interventions, the physical functioning domain was substantially modulated by the exercise protocols.

For physical functioning assessed via the SF-36, the 12-month aerobic and strength training program reported by Baglia *et al*. induced a statistically superior increase in physical scores in the IG compared with the CG (6.3 vs -1.1)^34^ and Paulo *et al*. reported strong time x group interactions for physical functioning (*p* = 0.008), with a high standardized effect size. However, physical functioning on the EORTC QLQ-C30 did not show significant differences^37^.

Liao *et al*. observed a significant improvement in EORTC QLQ-C30 physical functioning in the IG after the Baduanjin intervention (8.48 vs -3.66), with a large effect size^36^. On the other hand, the mat Pilates intervention did not result in significant changes in SF-36 functional capacity or EORTC QLQ-C30 physical functioning^38^.

Finally, the combined exercise intervention by Baglia *et al*. showed significant differences in the FACT-G physical well-being and functional well-being subscales; in line with these findings, the SF-36 role-physical subscale was also significant^34^.

Following a health promotion intervention, Goodwin *et al*.^39^ reported significant improvement in physical functioning in the IG on both the SF-36 and the EORTC QLQ-C30. These findings were not replicated in the physiotherapy and hydrotherapy interventions^35^^,^^40^.

### Effects of Interventions on the Psychological Domain

The four studies evaluating physical exercise interventions^34^^,^^36^^-^^38^ yielded heterogeneous outcomes related to the psychological domain of QoL.

The 12-month aerobic and strength training program demonstrated significant improvements in the IG regarding SF-36 mental functioning (4.0 vs 0.7), but not regarding the FACT-G emotional well-being subscale^34^. The mat Pilates intervention reported a large effect size for improvement in the IG in both SF-36 mental health (7.0 vs 2.2) and EORTC QLQ-C30 emotional functioning, despite lacking statistical significance^38^. Conversely, the 9-month combined aerobic-resistance exercise program revealed no significant interaction effects or intergroup differences across any psychological dimensions, including the EORTC QLQ-C30 emotional functioning and SF-36 mental health subscales^37^. In contrast, the Baduanjin mind-body practice achieved significantly greater improvements in EORTC QLQ-C30 emotional functioning^36^.

Only the physiotherapy approach utilizing neuromuscular taping^35^ and the health promotion lifestyle intervention^39^ evaluated this domain. Neither strategy yielded statistically significant results related to the EORTC QLQ-C30 emotional functioning subscale or the SF-36 mental component scores^35^^,^^39^.

### Effects of Interventions on the Social Domain

Five studies assessed social functioning, comprising the four physical exercise intervention^34^^,^^36^^-^^38^ and the physiotherapy intervention^35^. Across all studies, this domain was assessed using the SF-36 and/or the EORTC QLQ-C30.

The SF-36 social functioning subscale scores increased significantly in the IG following the 12-month exercise programme (6.2 vs 1.4)^34^. This is consistent with the significant group × time interaction for the social dimension with a large effect size^37^^,^^38^. Conversely, when social functioning was assessed using the EORTC QLQ-C30, no statistically significant differences were observed for the remaining interventions^36^, nor were they captured by the studies that observed positive changes using the SF-36^37^^,^^38^.

The physiotherapy intervention using neuromuscular taping^35^ did not show significant differences in this domain (EORTC QLQ-C30).

### Effects of Interventions on the Spiritual Domain

Regarding spiritual aspects, the measurement instruments used focused primarily on the functional, symptomatic, and psychological domains, without reporting specific items for the construct of spirituality.

### Effects on Other Specific Aspects of Functioning

- *Cognitive functioning*: Three studies evaluating physical exercise interventions assessed EORTC QLQ-C30 cognitive functioning. The 12-week Baduanjin program showed significantly greater improvements in the IG (9.21 vs. -0.83)^36^. Conversely, cognitive functioning measures remained statistically unaltered, with no significant interaction effects over time observed in either the combined exercise training trial^37^ or the 24-week mat Pilates protocol^38^.
- *Role functioning*: Of the four studies that assessed EORTC QLQ-C30 role functioning^35^^-^^38^, only the Baduanjin training demonstrated significantly greater post-intervention improvements (6.88 vs. -1.29)^36^.
- Sexual functioning: Two studies provided data regarding EORTC QLQ-BR23 sexual function, with no statistically significant changes^37^^,^^38^.

To complement the detailed narrative synthesis presented above and facilitate an integrative interpretation of the current evidence, a summary of findings in shown in Table 2.

**Table 2:** Summary of findings for overall quality of life and domains, including the certainty of evidence (over 4) based on the authors’ judgement

QoL Domain Studies Size	Average methodological quality (Risk of bias)	Direction of effect and clinical impact. Effect size range (Cohen’s d / Hedges’ g)	Certainty of Evidence
Overall and general health perception 7^34^^-^^40^ n=713	Moderate overall: - Low Risk: n=4^34^^-^^37^ - Some Concerns: n=2^38^^,^^39^ - High Risk: n=1^40^ (unblinded outcome measurement)	Generally positive but highly instrument-dependent. Significant improvements captured by generic SF-36 (d=1.14 to 3.76)^37^^,^^38^, FACT-G/B (0.46)^34^, and breast cancer-specific EORTC QLQ-BR23 (p=0.0031)^40^. Conversely, core EORTC QLQ-C30 global scale showed non-significant results across most trials, except for Baduanjin (g=0.97)^36^ and physiotherapy intervention (neuromuscular taping) (g=1.73)^35^ Small to very large (0.46 to 3.76)	3-Moderate Downgraded by one level due to inconsistency across different measurements instruments (SF-36 vs. EORTC QLQ-C30)
Physical 6^34^^-^^39^ n=643	High to Moderate overall: - Low Risk: n=4^34^^-^^37^ - Some Concerns: n=2^38^^,^^39^	Highly positive and clinically significant across physical exercise and health promotion interventions: - A 12-month aerobic/strength program significantly improved SF-36 physical scores (p<0.0001) and FACT-G physical/functional well-being (p≤0.002)^34^. Large and significant effects were reported in a 9-month combined training (SF-36: d=1.32)^37^, Baduanjin (EORTC QLQ-C30: g=1.03)^36^, and a telephone-based health promotion trial (d=0.22 for both SF-36 and EORTC QLQ-C30)^39^ - Mat Pilates ^38^ and neuromuscular taping physiotherapy^35^ showed no statistically significant changes in physical functioning Small to very large (d/g=0.22 to 1.32).	3-Moderate Downgraded by one level due to inconsistency
Psychological 6^34^^-^^39^ n=643	High to Moderate overall: - Low Risk: n=4^34^^-^^37^ - Some Concerns: n=2^38^^,^^39^	Highly heterogeneous and inconsistent across interventions: - Positive impacts: A 12-month aerobic/strength program significantly improved SF-36 mental functioning (p=0.01, but showed null effects on FACT-G emotional well-being)^34^. Mat Pilates led to significant SF-36 mental health improvements (p=0.026, d=1.56) and a large clinical effect in EORTC QLQ-C30 emotional functioning (d=1.71, though non-significant)^38^. Baduanjin also achieved significantly greater improvements in EORTC QLQ-C30 emotional functioning (p=0.010)^36^ - No significant changes were found following the 9-month combined exercise program^37^, the neuromuscular taping physiotherapy^35^, or the health promotion lifestyle intervention^39^ across any psychological subscales Moderate to very large (*d*=1.56 to 1.71)	3-Moderate Downgraded by one level due to inconsistency
Social 5^34^^-^^38^ n=309	High to Moderate overall: - Low Risk: n=4^34^^-^^37^ - Some Concerns: n=1^38^ (randomization uncertainties or minor deviations)	Inconsistent results based on the measurement instrument used: - Significant improvements and large clinical impacts were captured by the SF-36 subscale following a 12-month program (p<0.001)^34^, a 9-month combined training (d=1.10)^37^, and mat Pilates (d=2.49)^38^ - Conversely, no significant differences were observed across any intervention when evaluated via the EORTC QLQ-C30^36^, including the physiotherapy (neuromuscular taping) trial^35^ Large to very large (d=1.10 to 2.49)	3-Moderate Downgraded by one level due to inconsistency across different QoL measurement instruments (SF-36 vs. EORTC QLQ-C30)
Spiritual 0 -	-	-	-

QoL: Quality of Life. EORTC-QLQ-C30: European Organization for the Research and Treatment of Cancer Quality of Life Questionnaire - Core 30; EORTC-QLQ-BR23: European Organization for the Research and Treatment of Cancer Quality of Life Questionnaire - Breast Cancer; FACT-G: Functional Assessment of Cancer Therapy - General; FACT-B: Functional Assessment of Cancer Therapy - Breast; SF-36: 36-Item Short Form Survey.

## DISCUSSION

This systematic review aimed to identify, evaluate, and synthesize the available scientific evidence regarding the impact of non-pharmacological interventions on QoL and its specific domains (physical, psychological, social, and spiritual) in female BCS undergoing ET. The results reveal that structured physical exercise-based strategies are the most consistent and effective non-pharmacological interventions for improving QoL in this population.

While other non-pharmacological approaches, such as physiotherapy using neuromuscular taping, specialized hydrotherapy, and health-promotion interventions, provide localized or specific symptom relief, exercise-based interventions yield the most comprehensive and systemic improvements. These benefits are broadly observed across the physical, psychological, and social domains, mitigating several of the most debilitating side effects associated with ET. Notably, despite the comprehensive nature of our objective, evidence reveals a significant gap regarding the spiritual domain, as the standardized questionnaires used in the included trials focus exclusively on functional, symptomatic, and psychosocial domains without incorporating specific items to measure spirituality. As Firkins *et al*. (2025)^41^ highlight, generic tools often measure functional well-being rather than holistic QoL. Consequently, the spiritual benefits of interventions may remain undetected. Future trials should incorporate specific instruments, such as the FACIT-Sp, to accurately evaluate this domain^41^.

Our findings align with broader evidence on the health benefits of physical activity in survivorship care. Recent systematic reviews confirm that physical exercise interventions are effective strategies that positively affect health-related QoL, cardiorespiratory fitness, and body composition by mitigating treatment-related adverse events^42^. Furthermore, the latest National Comprehensive Cancer Network Guidelines (Version 2.2026) explicitly endorse maintaining an active lifestyle and an ideal body weight to optimize BC outcomes^43^.

However, the impact of these interventions varies significantly depending on the assessed domain and modality. Regarding combined aerobic resistance training, this review corroborates findings from previous literature, such as the systematic review by Chan *et al*. (2020), who suggest that the intensity and duration of exercise protocols are crucial factors explaining discrepancies in QoL outcomes^28^. Long-term combined exercise programs consistently yield systemic improvements and represent a key component for managing the side effects associated to aromatase inhibitors.

On the other hand, mind-body interventions emerged as a comprehensive and efficient strategy. Baduanjin, in particular, achieved robust benefits in a shorter period^36^. It was the only intervention that generated significant improvements in the cognitive and social functioning domains. As corroborated by emerging literature, traditional mind-body practices like Baduanjin and Tai Chi integrate physical movement with meditation, rhythmic breathing, and relaxation techniques. This dual action of physical conditioning and psychological stabilization may interrupt the debilitating cycle of interconnected symptoms, providing comprehensive support for cancer survivors^44^. Baduanjin training has also been shown to improve physical function, mental health, and cognitive ability, and to reduce the risk of chronic diseases^45^. In relation to BC, a multicentre single-arm trial including patients with BC who participated in a 12-week supervised exercise intervention, reported improvements in HRQoL and symptoms^46^. A meta-analysis also reported that Tai Chi and Baduanjin significantly improve QoL indicators^44^.

A central finding of this systematic review is the profound heterogeneity within the existing literature, which manifests across three main areas: the interventions, the measurement tools, and the CG.

First, the diversity of non-pharmacological interventions reflects a wide range of therapeutic approaches. Many of these interventions combine multiple active elements, classifying them as complex interventions^47^. The inherent difficulty in designing, standardizing, and evaluating complex intervention may explain why overall effectiveness remains highly dependent on specific protocol designs.

Second, substantial heterogeneity was observed in the measurement instruments. Specific cancer-specific tools were used in the trials, including EORTC QLQ-C30^35^^,^^37^^,^^39^, FACT^34^, andEORTC QLQ-BR23^37^^,^^40^, as well as generic measurement instruments such as the SF-36^34^^,^^37^^,^^39^. Furthermore, in several studies, only certain domains or isolated items were analysed rather than global scores, which limits direct comparability between studies and precludes the performance of a formal quantitative meta-analysis.

Third, the synthesis of the studies revealed two methodological approaches regarding the CG. While several trials used passive usual care as a comparator^34^^,^^36^^,^^40^, a substantial proportion featured active controls or sham placebos^35^^,^^37^^-^^39^. This diversity fundamentally influences the interpretation of the results. Comparing interventions with passive controls tends to maximize differences in QoL scores because of the contrast with sedentary behaviour. Conversely, active controls account for the *attention effect*, narrowing the differences between groups and providing a more conservative and clinically realistic estimate of the therapeutic impact. This heterogeneity must be carefully considered when generalizing the efficacy of these strategies.

The variability of these results suggests that non-pharmacological interventions are not interchangeable. For instance, while the health promotion intervention^39^ may successfully target physical function, the physiotherapy intervention^35^ addresses localized pain and fatigue. Consequently, clinical prescription should be specific and tailored to the patient’s primary baseline complaint.

Finally, not all interventions had improving QoL as their primary objective. Some focused on specific effects and considered QoL assessment as a secondary outcome^36^^,^^37^^,^^40^. Thus, interventions designed to address specific effects may have a limited impact on QoL^39^. Only two studies considered the effect on QoL as the primary outcome^37^^,^^38^.

This study presents a systematic review with considerable methodological rigor and an exhaustive search of the scientific literature. However, several limitations within the available evidence must be addressed. Regarding the literature search, the strategy was restricted to the main databases over the last 13 years and to specific languages, without including clinical trial registries or grey literature sources, which may have resulted in the exclusion of relevant studies. Another significant limitation is that some of the included trials evaluated QoL as a secondary outcome. In these cases, sample sizes were not calculated based on QoL, which may limit the statistical power to detect subtle changes and requires cautious interpretation of their effects. Finally, regarding the pooled evidence, the overall strength of our conclusions is limited by the small number of included studies and the specific risks of bias identified in several trials, as well as by the current absence of a formal GRADE certainty assessment.

In conclusion, this systematic review highlights the scarcity of interventions specifically aimed at improving the QoL in female BCS undergoing ET, as well as the marked heterogeneity among the available studies. Non-pharmacological interventions, particularly certain modalities of physical exercise, show potential benefits; however, the overall effects on QoL remain inconsistent, and the evidence is limited by substantial methodological diversity. Our findings indicate that the effectiveness of interventions varies according to their design and the QoL domains targeted, underscoring that interventions are not interchangeable.

Considering the multiple side effects associated with the use of ET, future research should strive to improve methodological standardization and reduce data variability by testing consistent frequencies, intensities, and durations, to enable more definitive conclusions. Ultimately, exploring strategies to increase long-term adherence will be crucial to ensuring sustained improvements in QoL in female BCS undergoing ET and may ultimately contribute to reducing the risks of recurrence and mortality.

## APPENDIX I

**Table:** Search strategies

Data bases	Search strategies
PubMed	(“Cancer Survivors”[Mesh] OR “cancer survivors*” OR “cancer survivorship” OR “breast cancer survivor*”) AND (“Breast Neoplasms”[Mesh] OR “breast neoplasm*” OR “breast tumor*” OR “breast cancer*” OR “breast carcinoma*”) AND (“Quality of Life”[Mesh] OR “quality of life” OR “Life Quality” OR “Health-Related Quality Of Life” OR “HRQOL”) AND (“Hormone Replacement Therapy”[Mesh] OR “hormone replacement therapy” OR “hormone therapy” OR “endocrine therapy” OR “tamoxifen” OR “aromatase inhibitor therapy” OR “letrozole” OR “anastrozole” OR “exemestane”)
Cochrane	#1 MeSH descriptor: [Cancer Survivors] explode all trees
	#2 cancer* NEXT survivors* OR “cancer survivorship” OR breast* NEXT cancer* NEXT survivor*
	#3 #1 OR #2
	#4 MeSH descriptor: [Breast Neoplasms] explode all trees
	#5 breast* NEXT neoplasm* OR “breast tumor” OR “breast cancer” OR “breast carcinoma”
	#6 #4 OR #5
	#7 MeSH descriptor: [Quality of Life] explode all trees
	#8 “quality of life” OR “Life Quality” OR “Health Related Quality Of Life” OR “HRQOL”
	#9 #7 OR #8
	#10 MeSH descriptor: [Hormone Replacement Therapy] explode all trees
	#11 “hormone therapy” OR “endocrine therapy” OR “tamoxifen” OR “aromatase inhibitor therapy” OR “letrozole” OR “anastrozole” OR “exemestane”
	#12 #10 OR #11
	#13 #3 AND #6 AND #9 AND #12
Scopus	TITLE-ABS-KEY ( ( (“cancer survivor*” OR “cancer survivorship” OR “breast cancer survivor*” ) AND (“breast neoplasm*” OR “breast tumor*” OR “breast cancer*” OR “breast carcinoma*”) AND (“Quality of Life” OR “Life Quality” OR “health related quality of life” OR “HRQOL”) AND (“Hormone Replacement Therapy” OR “hormone therapy” OR “endocrine therapy” OR “tamoxifen” OR “aromatase inhibitor therapy” OR “letrozole” OR “anastrozole” OR “exemestane”) ) ) AND (LIMIT-TO (DOCTYPE, “ar” ) )
Web of Science	#1 AB=(((cancer survivor OR cancer survivorship OR breast cancer survivor) AND (breast neoplasm OR breast tumor OR breast cancer OR breast carcinoma) AND (quality of life OR Life Quality OR Health-Related Quality Of Life OR Health Related Quality Of Life OR HRQOL) AND (hormone replacement therapy OR hormone therapy OR endocrine therapy OR tamoxifen OR aromatase inhibitor therapy OR letrozole OR anastrozole OR exemestane)))
	#2 TI=(((cancer survivor OR cancer survivorship OR breast cancer survivor) AND (breast neoplasm OR breast tumor OR breast cancer OR breast carcinoma) AND (quality of life OR Life Quality OR Health-Related Quality Of Life OR Health Related Quality Of Life OR HRQOL) AND (hormone replacement therapy OR hormone therapy OR endocrine therapy OR tamoxifen OR aromatase inhibitor therapy OR letrozole OR anastrozole OR exemestane)))
	#3 #2 OR #1
